# Effective Inhibition of Human Immunodeficiency Virus 1 Replication by Engineered RNase P Ribozyme

**DOI:** 10.1371/journal.pone.0051855

**Published:** 2012-12-26

**Authors:** Wenbo Zeng, Yuan-Chuan Chen, Yong Bai, Phong Trang, Gia-Phong Vu, Sangwei Lu, Jianguo Wu, Fenyong Liu

**Affiliations:** 1 State Key Laboratory of Virology, College of Life Sciences, Wuhan University, Wuhan, Hubei, China; 2 Program in Comparative Biochemistry, University of California, Berkeley, California, United States of America; 3 School of Public Health, University of California, Berkeley, California, United States of America; Temple University School of Medicine, United States of America

## Abstract

Using an in vitro selection procedure, we have previously isolated RNase P ribozyme variants that efficiently cleave an mRNA sequence in vitro. In this study, a variant was used to target the HIV RNA sequence in the *tat* region. The variant cleaved the tat RNA sequence in vitro about 20 times more efficiently than the wild type ribozyme. Our results provide the first direct evidence that combined mutations at nucleotide 83 and 340 of RNase P catalytic RNA from *Escherichia coli* (G_83_ -> U_83_ and G_340_ -> A_340_) increase the overall efficiency of the ribozyme in cleaving an HIV RNA sequence. Moreover, the variant is more effective in reducing HIV-1 p24 expression and intracellular viral RNA level in cells than the wild type ribozyme. A reduction of about 90% in viral RNA level and a reduction of 150 fold in viral growth were observed in cells that expressed the variant, while a reduction of less than 10% was observed in cells that either did not express the ribozyme or produced a catalytically inactive ribozyme mutant. Thus, engineered ribozyme variants are effective in inhibiting HIV infection. These results also demonstrate the potential of engineering RNase P ribozymes for anti-HIV application.

## Introduction

Human Immunodeficiency Virus (HIV) is the etiological agent of Acquired Immunodeficiency Syndrome (AIDS) [1], [2]. Combinations of antiviral agents, such as highly active antiretroviral therapy (HAART), have significantly suppressed levels of plasma viral loads with improved survival and outcomes of patients infected with HIV [3], [4]. However, with the issues of the emergence of viral escape mutants, significant drug side effects and strict patient compliance, HAART remains problematic [5], [6]. Therefore, there is an obvious need to consider other additional therapeutic options such as gene therapy [7], [8]. Continued development of new antiviral compounds and novel approaches is central for the treatment and prevention of HIV infection and AIDS.

Nucleic acid-based gene interference technologies, including conventional antisense molecules, aptamers, ribozymes, and small interfering RNAs (siRNAs), represent promising gene-targeting strategies for specific inhibition of mRNA sequences of choice. [8], [9], [10]. For example, siRNAs, which can be either expressed endogenously or administered exogenously, induce the endogenous RISC RNases of the RNA interference (RNAi) pathway, leading to cleavage of a specific mRNA [8], [11]. The siRNA-based approach has been shown to be extremely effective in blocking gene expression and replication of human viruses including HIV and HCMV while aptamers can also be used as a class of promising therapeutic agents for anti-HIV applications [10], [12], [13], [14], [15], [16].

RNA enzymes are also being developed as promising gene-targeting reagents [17], [18], [19], [20], [21]. Compared to conventional antisense and RNAi molecules, a ribozyme may have several unique features. For example, ribozymes, like aptamers, are much less likely than RNAi molecules to saturate cellular factors required for their processing, such as Exportin V, Drosha, or Dicer [8], [9], [10], [16]. Furthermore, a ribozyme can be easily engineered to improve its catalytic activity and specificity while its expression and delivery into specific cellular compartments can be manipulated. Both hammerhead and hairpin ribozymes have been shown to cleave viral mRNA sequences and inhibit viral replication in cells infected with HIV-1 while a ribozyme derived from a group I intron has been used to repair mutant mRNAs in cells [17], [18], [20]. Thus, ribozymes can be used as a tool in both basic and clinical research, such as in studies of developmental processes and in antiviral gene therapy [9], [22].

**Figure 1:**
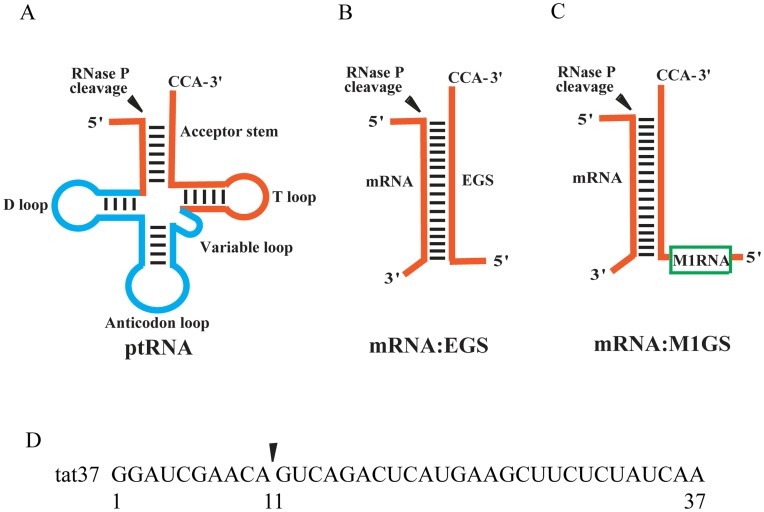
Schematic representation of a natural substrate (ptRNA)(A), a small model substrate (EGS:mRNA) for ribonuclease P and M1 RNA from *E. coli* (B), and a complex formed between a M1GS RNA and its mRNA substrate (C). A part of the EGS:mRNA and M1GS:mRNA complexes resembles the acceptor stem and T-stem domains of a ptRNA (shown in bold type). The site of cleavage by RNase P or M1 RNA is marked with a filled arrow. (D) Schematic representation of the substrate used in the study. The targeted sequences that bind to the guide sequences of the ribozymes are highlighted.

Ribonuclease P (RNase P) is a ribonucleoprotein complex responsible for the maturation of the 5′ termini of tRNAs [23], [24], [25]. In bacteria, the RNase P holoenzyme contains a catalytic RNA subunit and a small basic protein subunit (e.g. M1 RNA and C5 protein in *Escherichia coli*) [26]. One of the unique features of the RNase P holoenzyme and its catalytic RNA is their ability to recognize the structures, rather than the sequences of their substrates, which gives them the ability to hydrolyze different substrates [23], [24], [25]. Thus, M1 ribozyme can cleave an mRNA substrate as long as the target sequence hybridizes with its complementary sequence (designated as external guide sequence or EGS) to form a complex resembling the portion of a tRNA molecule that includes the acceptor stem, the T-stem, the 3′ CCA sequence, and the 5′ leader sequence (Figure 1) [27], [28]. A sequence-specific ribozyme, M1GS RNA, can be constructed by covalently linking a guide sequence to the 3′ terminus of M1 RNA (Figure 1) [19], [29]. M1GS ribozymes have been constructed and used to inhibit the expression of both cellular genes and genes of herpes simplex virus 1 (HSV-1) and human cytomegalovirus (HCMV) [30], [31], [32]. Moreover, a reduction of 1000 fold in HSV-1 growth and a reduction of 150 fold in HCMV replication were observed in cells that expressed ribozymes derived from the wild type RNase P ribozyme sequence [30], [31]. It has previously been shown that custom-designed EGS molecules could induce human RNase P to cleave HIV RNA sequence in vitro and could inhibit HIV infection in cultured cells [33], [34]. However, it has not been reported whether M1GS ribozymes can block HIV infection in human cells.

Compared to hammerhead and hairpin ribozymes, RNase P ribozymes may possess several unique features as a gene targeting tool. For example, RNase P ribozymes (e.g. M1 RNA or M1GS RNA) can interact with specific cellular factors, which include the protein subunits of RNase P [24], [35]. Their activities can be stimulated significantly in the presence of cellular proteins. Therefore, RNase P ribozymes may be less susceptible to degradation by intracellular RNases and function more efficiently in the presence of cellular proteins [24], [35]. Targeted cleavage of mRNA by RNase P ribozyme provides a unique approach to inactivate any RNA of known sequence expressed *in vivo*. However, it has not been demonstrated whether RNase P ribozymes are also effective in inhibiting HIV-1 gene expression and replication. Meanwhile, increasing M1GS RNA catalytic efficiency *in vitro* and its efficacy *in vivo* is required in order to develop this ribozyme for practical use both as a research tool and as a therapeutic agent for gene-targeting applications. Using an *in vitro* selection procedure, we have previously isolated M1GS ribozyme variants that are more efficient in cleaving a specific mRNA sequence than that derived from the wild type M1 RNA [36]. In this study, we used one of these ribozyme variants to target the HIV-1 RNA genome region in the *tat* gene, and investigated its activity in cleaving the target RNA sequence *in vitro* and its efficacy in inhibiting HIV-1 gene expression and growth in cultured cells. The *tat* gene encodes an essential viral transcription factor that is required for HIV-1 gene expression [1], [2]. Targeting of the *tat* region in the HIV-1 genome by the ribozyme is expected to cleave the *tat* mRNA and in addition, the viral genomic RNA, leading to inhibition of viral replication. Our results indicate that the constructed ribozyme variant, when transiently or stably expressed in human cells, is more effective in inhibiting HIV-1 replication than the ribozyme derived from the wild type M1 RNA sequence. A reduction of more than 150 fold in the level of cell-free supernatant HIV-1 p24 was observed in cells that expressed the ribozyme variant. These results provide the first direct evidence that RNase P ribozyme can be effective in inhibiting HIV-1 gene expression and growth and furthermore, demonstrate the feasibility of developing highly effective RNase P ribozyme variants for anti-HIV-1 application by using *in vitro* selection procedures.

## Materials and Methods

### Ribozyme and Substrate Constructs

Plasmid pV38, pFL117, and pC102 contain the DNA sequence coding for V38, M1 RNA, and mutant C102 driven by the T7 RNA polymerase promoter [36], [37]. Ribozyme variant 38 (designated as V38) contains two point mutations (i.e. G_83_ -> U_83_ and G_340_ -> A_340_) [36] while mutant ribozyme C102 contains several point mutations (e.g. A_347_C_348_ -> C_347_U_348_, C_353_C_354_C_355_G_356_ -> G_353_G_354_A_355_U_356_) at the catalytic domain (P4 helix) [37]. The DNA sequences that encode ribozymes V38-TAT, M1-TAT, and C-TAT were constructed by PCR using the DNA sequences of the ribozymes as the templates and oligonucleotides AF25 and RbGS-TAT (5′-CCCGCTCGAGAAAAAATGGTGTCAGACTCATCAAGCTTCGAGCTATGACCATG-3′) as 5′ and 3′ primers, respectively. The DNA sequence that encodes substrate tat37 was constructed by PCR using pGEM3zf(+) as a template and oligonucleotides AF25 (5′-GGAATTCTAATACGACTCACTATAG-3′) and sTAT (5′-TTGATAGAGAAGCTTGATGAGTCTGACTGTTCGATCCTATAGTGAGTCGTATTA-3′) as 5′ and 3′ primers, respectively.

### Cleavage and Binding Analysis

M1GS RNAs and the *tat* mRNA substrate (i.e. tat37) were synthesized *in vitro* by T7 RNA polymerase (Promega Inc. Madison, WI) following the manufacturer’s recommendations and further purified on 8% polyacrylamide gels containing 8 M urea. Subsequently, the M1GS RNAs were mixed with the [^32^P]-labeled RNA substrate. The cleavage reactions were carried out at 37°C in a volume of 10 µl for 30 min in buffer A (50 mM Tris, pH 7.5, 100 mM NH_4_Cl, and 100 mM MgCl_2_) [36]. Cleavage products were separated in denaturing gels and quantitated with a STORM840 phosphorimager.

Assays to determine the observed reaction rate (k_obs_) and the values of the cleavage efficiency [(k_cat_/K_m_)^s^] were performed under single turnover conditions as described previously [36], [37]. Briefly, we performed analyses with a trace amount of radioactive substrate and an excess of ribozyme. The concentration of radioactive substrate was less than 0.1 nM, and the concentrations of ribozyme tested ranged from 0.5 to 100 nM. Variants of the amount of substrate did not affect the observed cleavage rate (k_obs_) at a fixed excess ribozyme concentration and the reaction followed pseudo-first-order kinetics. We assayed pseudo-first-order rate constants of cleavage (k_obs_) at each ribozyme concentration by determining the slope of a plot −ln[(Ft−Fe)/(1−Fe)] versus time using Kaleidagraph (Synergy Software, Reading, PA). Ft and Fe represent the fraction of the substrate at time t and at the end point of the experiments, respectively. We calculated the values of the overall cleavage rate [(k_cat_/K_m_)^s^] by determining the slope of a least-squares linear regression (Kaleidagraph) of a plot of the values of k_obs_ versus the concentrations of the ribozymes. These values were the averages of five independent experiments.

The procedures to measure the equilibrium dissociation constants (K_d_) of the M1GS-tat37 complexes were modified from Pyle et al [38] and have been described previously [36]. In brief, various concentrations of ribozyme (0.0005–50 nM) were preincubated in buffer D (50 mM Tris, pH 7.5, 100 mM NH4Cl, 100 mM CaCl2, 3% glycerol, 0.1% xylene cyanol, 0.1% bromphenol blue) for 10 min before mixing with an equal volume of 1–10 pM substrate RNA preheated under identical conditions. The samples were incubated for 10–120 min to allow binding, then loaded on a 5% polyacrylamide gel, and run at 10 watts. The electrophoresis running buffer contained 100 mM Tris-Hepes, pH 7.5, and 10 mM MgCl2 [38]. The value of K_d_ was then extrapolated from a graph plotting percentage of product bound *versus* ribozyme concentration. The values were the averages of five independent experiments.

### Studies of Antiviral Activity of Transiently-expressed Ribozymes

The DNA sequences coding for V38-TAT, M1-TAT, and C-TAT were cloned into retroviral vector LXSN and placed under the control of the small nuclear U6 RNA promoter, which has previously been shown to express M1GS RNA and other RNAs steadily [30], [39]. 293T cells were trypsinized and plated at 1×10^5^ cells per well in six-well plates. 24 hours later, the LXSN-M1GS DNA was transfected alone or co-transfected with the pHIV_NL4-3_ DNA (obtained from the NIH AIDS Research and Reference Program) using Lipofectamine (Invitrogen, San Diego, CA). Forty-eight hours post transfection, both the transfected cells and culture media were harvested. Cell-free supernatants were isolated from the culture media and viral production was quantified by assaying the levels of HIV-1 p24 in the supernatants with a p24 ELISA kit (Beckman-Coulter, Fullerton, CA). RNA and protein samples were isolated from the transfected cells and the levels of total (unspliced and spliced) intracellular HIV RNA and viral p24 were determined by a real-time PCR assay and Western analysis, respectively.

### Studies of Antiviral Activity of Stably-expressed Ribozymes

The protocols for the construction of cells expressing different ribozymes were modified from Miller and Rosman [30], [39]. In brief, amphotropic PA317 cells were transfected with retroviral vector DNAs (LXSN-M1-TAT, C-TAT, and V38-TAT) with the aid of a mammalian transfection kit purchased from Invitrogen (San Diego, CA). Forty-eight hours post transfection, culture supernatants that contained retroviruses were collected and used to infect human H9 cells (obtained from the NIH AIDS Reagent and Reference Program). At 48–72 hours postinfection, cells were incubated in culture medium that contained 800 µg/ml neomycin. Cells were subsequently selected in the presence of neomycin for three weeks and neomycin-resistant cells were cloned.

To study the antiviral activity of the ribozymes, H9 cells and the constructed ribozyme-expressing cells were infected with strain HIV_NL4-3_ at a multiplicity of infection (MOI) of 0.02–0.1. At different time points of infection, the infected cells and culture media were harvested. The infected cells were used to determine the levels of total HIV RNA and p24, while the culture media were used to determine the levels of cell-free-supernatant HIV p24.

### Studies of the Expression of Ribozymes, Total Intracellular HIV RNA, and p24

For Northern analyses of the expression of the ribozymes, RNA fractions from M1GS-expression cells were isolated as described previously [30]. The RNA fractions were separated in a 2.5% agarose gel that contained formaldehyde, transferred to a nitrocellulose membrane, hybridized with the [^32^P]-radiolabeled DNA probes that contained the DNA sequence coding for M1 RNA and H1 RNA, respectively, and finally analyzed with a STORM840 phosphorimager. The radiolabeled DNA probe used to detect M1GS RNAs and H1 RNA was synthesized from plasmid pFL117 and pH1, by using a random primed labeling kit (Roche Applied Sciences, Indianapolis, IN).

For real-time PCR analyses of total intracellular HIV RNA, total cellular RNA samples were isolated from the cells that transiently or stably expressed the ribozymes, using Trizol reagent (Invitrogen, San Diego, CA), and treated with RQ1 DNase (Promega, Madison, WI). The treated RNA was reverse-transcribed in the presence of Powerscript reverse transcriptase (Clontech, Palo Alto, CA) using RT primer (5′-TTCCTGCCATAGGAGATGC-3′). The resulting cDNA (2 µl) was added to 48 µl of PCR mix containing 1×titanium Taq PCR buffer, 1 mM dNTPs, SYBR Green (1∶50,000), 10 nM fluorescein, 1×titanium Taq DNA polymerase (Clontech), and 20 pmol each of 5′ primer HIV5 (5′-CATCCAGGAAGTCAGCCT-3′) and 3′ primer HIV3 (5′-TTCCTGCCATAGGAGATGC-3′). To normalize the RNA level, the level of actin mRNA was assayed by real-time PCR using the same PCR mix except for the primers which were Actin5 (5′-TGACGGGGTCACCCACACTGTGCCCATCTA-3′) and Actin3 (5′-CTAGAAGCATTGCGGTGGCAGATGGAGGG-3′), respectively [40].

Real-time PCR was carried out in an iCycler (Bio-Rad, Hercules, CA) and the PCR consisted of 35 cycles with denaturation at 94°C for 40 seconds, followed by primer annealing at 50°C for 40 seconds and extension at 72°C for 40 seconds. We also generated a standard (dilution) curve by amplifying different dilutions of the RNA transcript of the *tat* sequence that was produced by an in vitro transcription kit with T7 RNA polymerase (Promega, Madison, WI). The real-time PCR results were derived from three independent experiments.

Western analyses were performed to determine the intracellular levels of HIV p24 protein. The polypeptides from cell lysates were separated on either 7.5% or 9% [vol/vol] SDS-polyacrylamide gels cross-linked with *N*, *N*’-methylenebisacylamide, transferred electrically to nitrocellulose membranes. We stained the membranes using the antibodies against HIV p24 and human actin in the presence of a Western chemiluminescent substrate kit (GE Healthcare) with an alkaline phosphatase-conjugated antibody, and analyzed the stained membranes with a STORM840 phosphorimager. The anti-p24 and anti-actin monoclonal antibodies were obtained from the NIH AIDS Research and Reference Program or purchased from Sigma (St Louis, MO), respectively. Quantification was performed in the linear range of protein detection.

To determine the p24 level in the cell-free supernatants, the supernatants were isolated from the culture media collected from cultures of cells that transiently or stably expressed the ribozymes and were infected with HIV-1. The levels of HIV-1 p24 was assayed by a p24 ELISA kit (Beckman-Coulter, Fullerton, CA).

## Results

### In vitro Characterization of the Cleavage of HIV-1 tat RNA Sequence by the Constructed RNase P Ribozymes

Most mRNA species inside cells are believed to be associated with proteins and are present in a highly organized and folded conformation. Thus, it is important to choose a targeting region that is accessible to binding of ribozymes in order to achieve efficient cleavage. Using dimethyl sulphate (DMS), we employed an *in vivo* mapping approach [19], [41] to determine the accessibility of the region of the *tat* RNA in HIV-1-infected cells. A position, 30 nucleotides upstream from the 3′ terminus of the exon 1 of the tat mRNA [1], [42], was chosen as the cleavage site for M1GS RNA. This region is among the most accessible to DMS modification, and presumably to ribozyme binding. Furthermore, this site appears to be one of the regions that is highly conserved among HIV genome sequences [42].

Using an *in vitro* selection procedure, we have isolated M1GS ribozyme variants that are more efficient in cleaving the mRNA sequence (TK mRNA) encoding the HSV-1 thymidine kinase (TK) than the ribozyme derived from the wild type M1 RNA sequence [36]. However, whether these ribozyme variants can be used and are highly effective in inhibiting HIV-1 gene expression and replication has not been demonstrated. We chose variant 38 (designated as V38) for the study because the ribozymes derived from this variant are among the most active M1GS RNAs in cleaving the TK mRNA as well as the *tat* RNA sequence (see below, Table 1). This variant contains two point mutations (i.e. G_83_ -> U_83_ and G_340_ -> A_340_) [36].

**Table 1:** Overall cleavage rate [(k_cat_/K_m_)^s^] and binding affinity (K_d_) in cleavage reactions of tat37 with RNase P ribozymes.

Enzyme	(k_cat_/K_m_)^s^ (µM^−1^·min^−1^)	K_d_ (nM)
M1-TAT	0.30±0.12	0.25±0.07
V38-TAT	6.2±2.1	0.22±0.06
C-TAT	<5×10^−5^	0.21±0.06

Binding assays were carried out in buffer D (50 mM Tris, pH 7.5, 100 mM NH4Cl, 100 mM CaCl2, 3% glycerol, 0.1% xylene cyanol, 0.1% bromphenol blue), using a protocol modified from Pyle et al [38]. Single-turnover kinetic analyses to determine the values of (k_cat_/K_m_)^s^ were carried out in buffer A (50 mM Tris-HCl, pH 7.5, 100 mM NH_4_Cl, 100 mM MgCl_2_) as described previously [36]. The values shown are the averages derived from five independent experiments.

**Figure 2:**
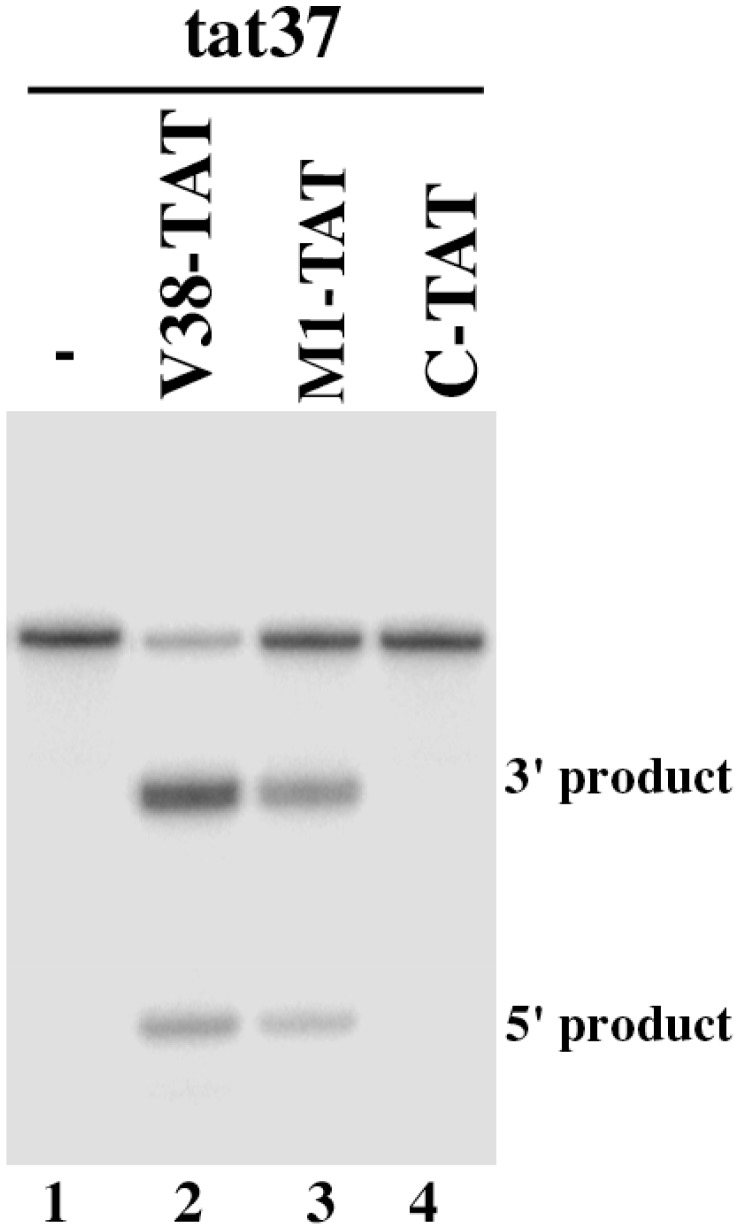
Cleavage of substrate tat37 by M1GS RNA. Substrate (20 nM) was incubated alone (lanes 1), with 1 nM of V38-TAT (lane 2), 5 nM of M1-TAT (lanes 3), or 5 nM of C-TAT ribozyme (lanes 4). Cleavage reactions were carried out for 30 min in buffer A (50 mM Tris^.^HCl, pH 7.5, 100 mM NH_4_Cl, 100 mM MgCl_2_) at 37°C. Cleavage products were separated in 15% polyacrylamide gels containing 8 M urea.

Ribozyme V38-TAT was constructed by covalently linking the 3′ terminus of V38 RNA with a guide sequence of 18 nucleotides that is complementary to the targeted *tat* RNA sequence. Two other M1GS ribozymes, M1-TAT and C-TAT, were also constructed in a similar way and were included in the study. M1-TAT was derived from the wild type M1 sequence. C-TAT was derived from C102 RNA, a M1 mutant that contained several point mutations at the catalytic P4 domain and was at least 10^4^-fold less active than M1 RNA in cleaving a pre-tRNA [37]. The DNA sequences coding for the M1GS ribozymes were generated by PCR using the DNA sequences for V38 RNA, M1 RNA, and C102 RNA as the templates and primers that contained the sequences complementary to the targeted *tat* RNA region. These DNA sequences were under the control of the promoter for T7 RNA polymerase and M1GS RNAs were synthesized *in vitro* from these DNA sequences by T7 RNA polymerase. A substrate, tat37, which contained the targeted *tat* RNA sequence of 37 nucleotides, was used (Figure 1B). In the absence of RNase P ribozymes (Figure 2, lane 1), no cleavage of the tat RNA sequence was detected. Efficient cleavage of the substrate by V38-TAT and M1-TAT was observed (Figure 2, lanes 2 and 3). In contrast, cleavage by C-TAT was barely detected (Figure 2, lane 4).

Using kinetic analyses, we determined the overall efficiency [measured as (k_cat_/K_m_)^s^] for these ribozymes in cleaving the *tat* RNA sequence substrate tat37. These results indicate that V38-TAT is about 20 fold more active than M1-TAT in cleaving tat37 (Table 1). Furthermore, we also carried out gel-shift assays to determine whether the differential cleavage efficiencies observed with V38-TAT, M1-TAT, and C-TAT were possibly due to their different binding affinities to the *tat* RNA sequence. Detailed assays under different concentrations of the ribozymes and tat37 indicate that the binding affinity of C-TAT to substrate tat37, measured as the dissociation constant (K_d_), is similar to those of M1-TAT and V38-TAT (Table 1). Since C-TAT contains the same antisense guide sequence and similar affinity to tat37 as V38-TAT and M1-TAT but is catalytically inactive, this ribozyme can be used as a control for the antisense effect in our experiments in cultured cells (see below).

### Transient Expression of RNase P Ribozymes for Inhibiting HIV-1 Gene Expression and Replication in Human Cells

**Figure 3:**
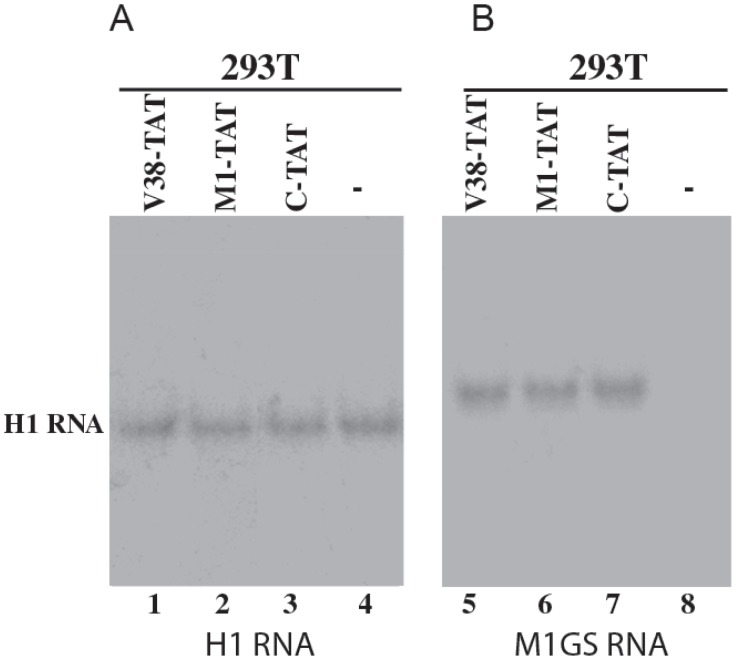
Northern analyses of the expression of M1GS ribozymes from the RNA fractions isolated from 293T cells that were transfected with pHIV_NL4-3_ alone (-, lanes 4 and 8) or from cells that were co-transfected with pHIV_NL4-3_ and a M1GS DNA construct to express V38-TAT (lanes 1 and 5), M1-TAT (lines 2 and 6), and C-TAT (lanes 3 and 7). Equal amounts of each RNA sample (25 µg) were separated on 2% agarose gels that contained formaldehyde, transferred to a nitrocellulose membrane, and hybridized to a [^32^P]-radiolabeled probe that contained the DNA sequence coding for M1 RNA (lanes 5–8) or H1 RNA (lanes 1–4), the RNA subunit of human RNase P [52]. The size of H1 RNA (∼380 nts) is very similar to that of M1GS RNA (∼450 nts) and therefore, can be used as a size marker. The expression of human H1 RNA was used as the internal loading control for the quantification of M1GS RNA expression. The hybridized products corresponding to the full-length retroviral transcripts (∼6 kb), transcribed from the LTR promoter, are at the top of the gel and are not shown.

The DNA sequences coding for V38-TAT, M1-TAT, and C-TAT were cloned into retroviral vector LXSN and placed under the control of the small nuclear U6 RNA promoter, which has previously been shown to express M1GS RNA and other RNAs steadily [28], [30], [39], [43]. This promoter is transcribed by RNA polymerase III, and its transcripts are highly expressed and primarily localized in the nucleus [28], [30], [43]. Human 293T cells were transfected with LXSN-M1GS DNAs. At 48 hours post-transfection, the transfected cells were harvested and total RNAs were isolated. The level of transient expression of the M1GS RNAs was investigated by Northern analysis of these RNA samples. M1GS RNAs were found to be expressed in these cells (Figure 3B) and the levels of the ribozymes were quantified using the expression level of human H1 RNA as the internal loading control (Figure 3A).

To determine whether M1GS RNAs can inhibit HIV-1 gene expression and replication in human cells that transiently expressed the ribozymes, 293T cells were co-transfected with various LXSN-M1GS constructs and a plasmid DNA construct containing the HIV-1_NL4-3_ sequence [42]. At 48 hours postinfection, both cells and culture medium supernatants were harvested. The expression of the ribozymes in these transiently transfected cells was detected and appeared to exhibit similar levels (Figure 3A–B). If the expression of these ribozymes blocks HIV-1 replication, it is expected that the levels of HIV-1 core antigen p24 and HIV-1 genomic RNA would be reduced. Accordingly, two series of experiments were carried out to determine the levels of HIV-1 gene expression and growth in the presence of M1GS RNA expression.

**Figure 4:**
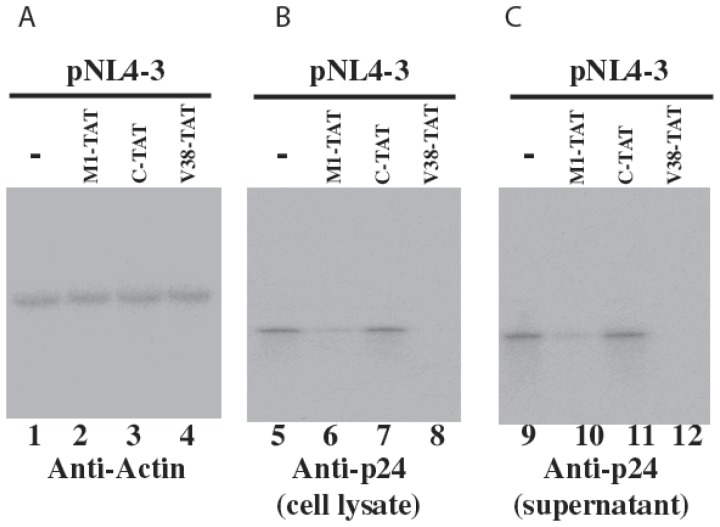
Expression of HIV p24 protein, as detected by Western blot analysis with a chemiluminescent substrate. Total protein fractions were isolated from 293T cell cultures that were transfected with pHIV_NL4-3_ alone (-; lanes 1, 5, and 9) or from cells that were co-transfected with pHIV_NL4-3_ and a M1GS DNA construct to express M1-TAT (lanes 2, 6, and 10), C-TAT (lines 3, 7, and 11), and V38-TAT (lanes 4, 8, and 12). Cells and culture media were harvested at 48 hours postinfection, and protein samples from cells (cell lysate, A–B) and cell-free supernatants (supernatant, C) were loaded on the gels. Equal amounts of protein samples (40 µg) isolated from cells were separated in SDS-polyacrylamide gels. The membranes were stained with the antibodies against human actin (A) and HIV p24 (B–C). The expression of human actin was used as the internal loading control for the quantification of HIV p24 expression.

**Figure 5:**
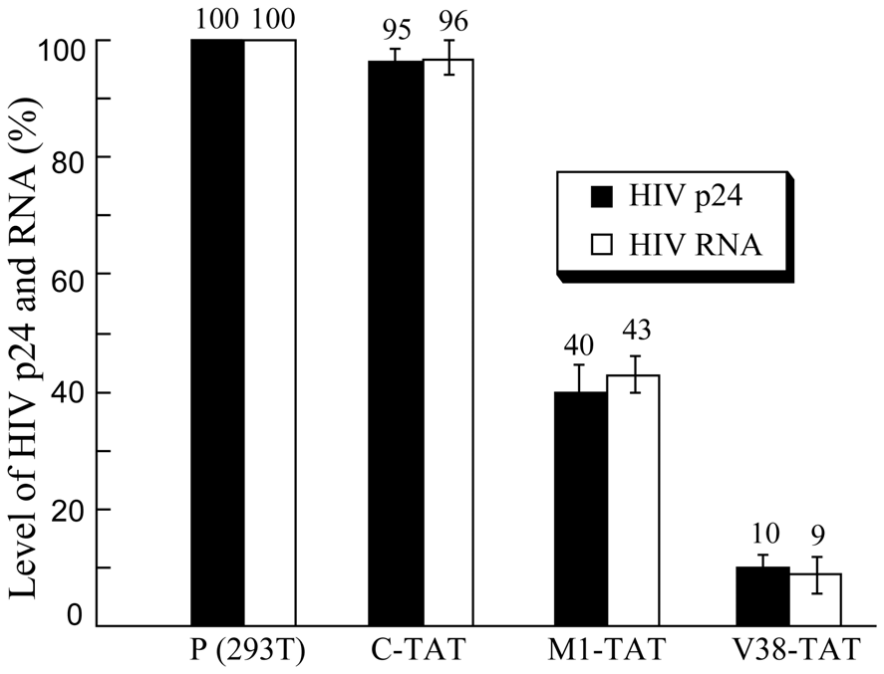
Schematic representation of the levels of supernatant HIV-1 p24 and total (unspliced and spliced) intracellular HIV RNA in pHIV_NL4-3_-transfected 293 T cells that did not express a ribozyme [P(293T)] or expressed ribozyme C-TAT (C-TAT), M1-TAT (M1-TAT), and V38-TAT (V38-TAT). The RNA and protein samples were isolated from cells and culture media at 48 hours postinfection, respectively. The levels of HIV RNA were determined using a real-time PCR assay while the levels of p24 protein were measured with a HIV p24 ELISA kit. The values shown are the averages from three independent experiments. The standard deviation is indicated by the error bars. Solid bars: HIV p24 protein; open bars: HIV RNA.

In the first series of experiments, Western analyses were carried out to determine the expression levels of HIV-1 p24. Protein samples were prepared from cell lysates and supernatants respectively, separated electrophoretically in SDS-polyacrylamide gels and electrically transferred to membranes. The membranes were stained with an anti-HIV-1 p24 antibody (anti-p24) and a monoclonal antibody against human actin (anti-actin) (Figure 4). The latter was used to detect the expression of actin that served as an internal loading control for the quantitation of HIV-1 p24 protein expression. A reduction of about 90%±5%, 58±5%, 6±3% (average of three experiments) in the level of intracellular HIV-1 p24 was observed in cells that expressed V38-TAT, M1-TAT, and C-TAT, respectively. The levels of p24 in the culture medium supernatants were also determined using an anti-p24 ELISA assay. We observed a reduction of about 90%±5%, 60±6%, 5±4% (average of three experiments) in the p24 level in culture media collected from cells that expressed V38-TAT, M1-TAT, and C-TAT, respectively (Figure 5). These results suggest that the subtantial reduction of HIV-1 p24 expression level in cells that expressed V38-TAT and M1-TAT was due to the targeted cleavage by the ribozymes. The low level of inhibition found in cells that expressed C-TAT was probably due to an antisense effect because C-TAT exhibited similar binding affinity to the target sequence as V38-TAT and M1-TAT but was catalytically inactive.

In the second series of experiments, we investigated the effect of transient expression of various M1GS RNAs on the levels of total (spliced and unspliced) HIV-1 intracellular RNA. RNA samples were isolated from cells at 48 hours post-transfection. The levels of total HIV-1 intracellular RNA were determined using a real-time PCR assay with the expression level of the actin mRNA as the internal control. The results of three independent experiments indicated that a reduction of 91%±7%, 57±6% and 4±3% in the level of total HIV-1 intracellular RNA was observed in cells that expressed V38-TAT, M1-TAT, and C-TAT, respectively (Figure 5). The low level of reduction in the level of HIV-1 RNA observed in cells that expressed C-TAT was presumably attributed to the antisense effect of the guide sequence.

### Stable Expression of RNase P Ribozymes for Blocking HIV-1 Production in Human Cells

To construct cell lines that express M1GS ribozymes, amphotropic packaging cells (PA317) [39] were transfected with LXSN-M1GS DNAs to produce retroviruses that contained the genes for M1GS RNA. Subsequently, human H9 cells, which were obtained from the NIH AIDS Reagent and Reference Program, were infected with these retroviruses, and cells expressing the ribozymes were cloned. The constructed lines and a control line in which cells were transfected with LXSN vector DNA alone were indistinguishable in terms of their growth and viability for up to two months (data not shown), suggesting that the expression of the ribozymes did not cause significant cytotoxicity.

**Figure 6:**
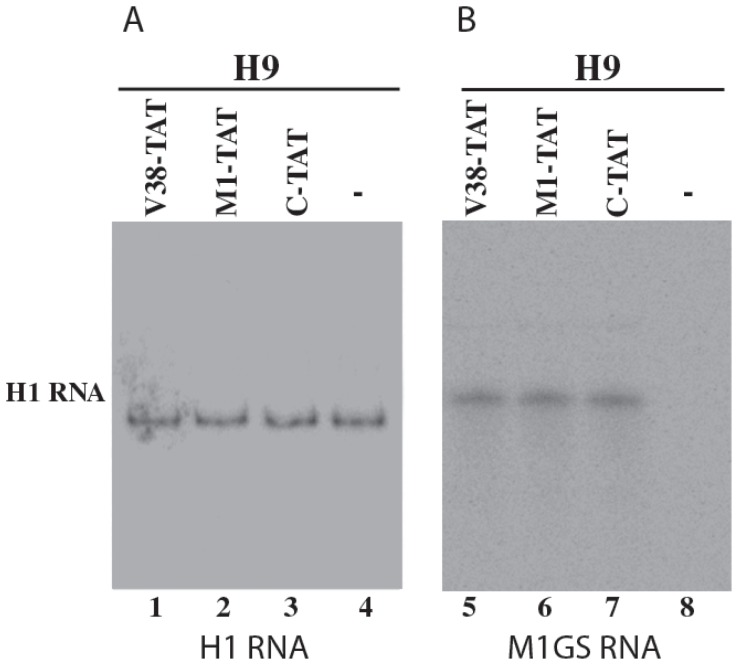
Northern analyses of the expression of M1GS ribozymes from the RNA fractions isolated from parental H9 cells (-, lane 4 and 8) or different cloned cell lines that expressed V38-TAT (lanes 1 and 5), M1-TAT (lanes 2 and 6), and C-TAT (lanes 3 and 7). Equal amounts of each RNA sample (25 µg) were separated on 2% agarose gels that contained formaldehyde, transferred to a nitrocellulose membrane, and hybridized to a [^32^P]-radiolabeled probe that contained the DNA sequence coding for H1 RNA (lanes 1–4) or M1 RNA (lanes 5–8). The expression of human H1 RNA was used as the internal loading control for the quantification of M1GS RNA expression.

The level of M1GS RNA in each cell clone was determined by Northern analysis with a DNA probe that is complementary to M1 RNA, using the expression of human H1 RNA as the internal loading control. Expression of the M1GS RNAs was detected in these cells (Figure 6). We only used the cell lines that expressed similar levels of these M1GS RNAs for further studies in tissue culture.

**Figure 7:**
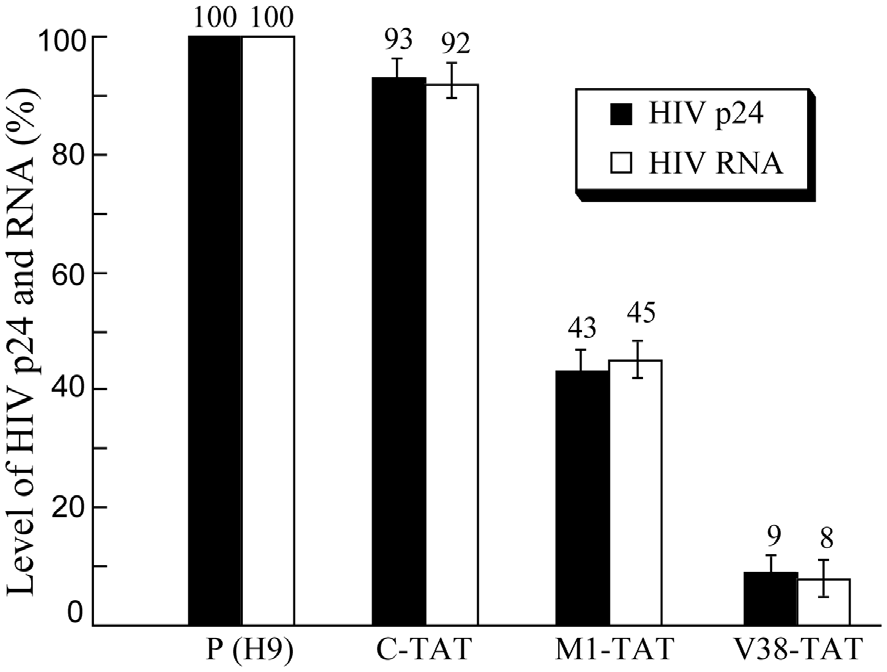
Schematic representation of the expression levels of supernatant HIV-1 p24 protein and total (unspliced and spliced) intracellular HIV RNA in viral infected H9 cells that did not express a ribozyme [P(H9)] or stably-expressed ribozyme C-TAT (C-TAT), M1-TAT (M1-TAT), and V38-TAT (V38-TAT). The values shown are the averages from three independent experiments. The standard deviation is indicated by the error bars. Solid bars: HIV p24 protein; open bars: HIV RNA.

To study the effect of stable expression of ribozymes on blocking HIV-1 production, cells were infected with HIV-1 at a multiplicity of infection (MOI) of 0.02–0.1 and three sets of experiments were carried out. First, supernatants were collected from cell cultures at 3 days postinfection, and the levels of p24 in the supernatants were determined using an anti-p24 ELISA assay. A reduction of about 91%±5%, 57±5%, 7±4% (average of three experiments) in the level of p24 was observed in cells that expressed V38-TAT, M1-TAT, and C-TAT, respectively (Figure 7). Since C-TAT binds to the target sequence as well as V38-TAT and M1-TAT but is catalytically inactive, our results suggest that the substantial reduction of p24 expression in cells that expressed V38-TAT and M1-TAT was due to the targeted cleavage by the ribozymes.

Second, we determined whether stable expression of M1GS ribozymes effectively reduces the level of total (spliced and unspliced) HIV-1 intracellular RNA. RNA samples were isolated from cells at 48–72 hours postinfection. Using the expression level of the actin mRNA as the internal control, we carried out a real-time PCR assay to determine the levels of total HIV-1 intracellular RNA. The results of three independent experiments indicated that a reduction of 92%±6%, 55±6% and 8±3% in the level of total HIV-1 intracellular RNA was observed in cells that expressed V38-TAT, M1-TAT, and C-TAT, respectively (Figure 7). The low level of reduction in the level of HIV-1 RNA observed in cells that expressed C-TAT was presumably due to the antisense effect of the guide sequence.

**Figure 8:**
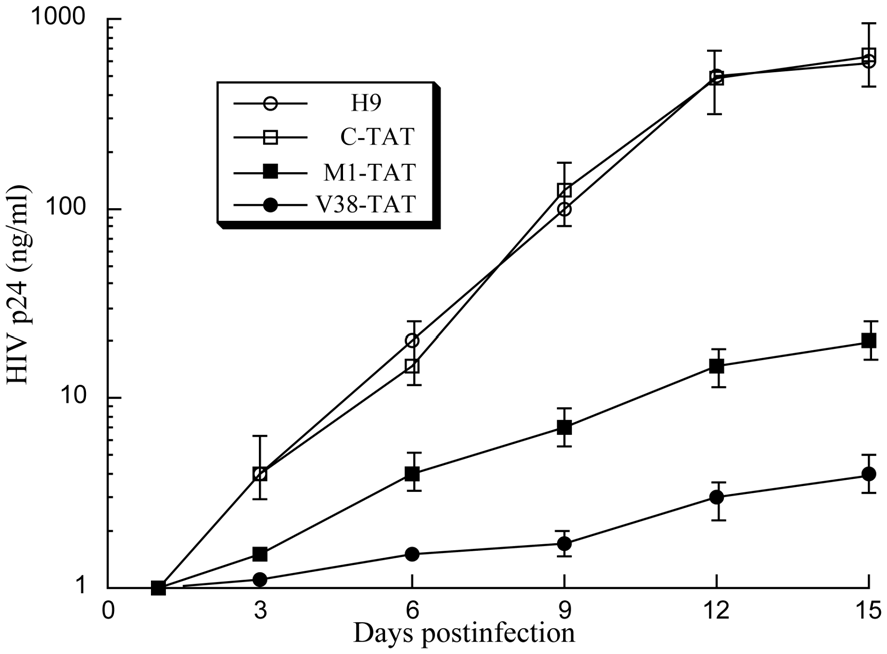
Growth of HIV-1 in H9 cells and cell lines that expressed M1GS RNAs. 5×10^5^ cells were infected with HIV-1 at a MOI of 0.02–0.1. Viral production was determined by a p24 antigen assay as a function of time postinfection. The parental H9 cells were used as a negative control. The values are the means from triplicate experiments. The standard deviation is indicated by the error bars.

The third set of experiments was carried out to study the magnitude and durability of the anti-HIV-1 effects of the stably-expressed ribozymes. The ribozyme-expressing cells were infected with HIV-1 at an MOI of 0.02–0.1. Cell-free supernatants containing newly produced viral particles were collected from the infected cultures at 3 day intervals through 15 days postinfection. The number of viral particles was determined by assaying the level of p24 antigen in the supernatants. The results of the experiments are summarized in Figure 8. After 12 days postinfection, a reduction of at least 150 and 30 fold in viral yield was observed in cells that expressed V38-TAT and M1-TAT, respectively, while no significant reduction was found in those that expressed C-TAT (Figure 8). These results are consistent with the notion that the cleavage of the *tat* RNA sequence in cells expressing V38-TAT and M1-TAT results in the observed reduction of the levels of HIV intracellular RNA and p24, and viral production.

## Discussion

Using ribozymes to inactivate mRNA of choice represents an appealing approach for gene therapy, since ribozyme cleavage of an mRNA target is highly specific and is irreversible [9], [22]. However, for M1GS ribozyme to be successful as a therapeutic tool, several criteria must be satisfied to achieve successful targeting. Among these are high efficiency of cleavage, sequence specificity of the ribozymes, and efficient delivery of the reagents.

Understanding the mechanism of ribozyme cleavage and improving its efficacy is essential to develop M1GS ribozyme for practical gene-targeting applications including anti-HIV therapy. However, little is currently known about the rate-limiting step of M1GS RNA cleavage reaction in cells. Equally unclear is whether the efficacy of the ribozymes can be improved, and if so, how it can be improved. In the present study, we showed that an RNase P variant, V38-TAT, exhibited at least 20 times higher rate of cleavage *in vitro* in cleaving the HIV-1 RNA genomic sequence than the ribozyme (i.e. M1-TAT) derived from the wild type M1 RNA sequence. Moreover, V38-TAT reduced the level of HIV total intracellular RNA in cultured cells by more than 90% and was more effective in cultured cells than M1-TAT, which decreased the viral RNA level by about 55–60%. A reduction of about 150 fold in HIV-1 production was observed in the V38-TAT-expressing cells while a reduction of about 30 fold was observed in M1-TAT-expressing cells. In contrast, a reduction of less than 10% in the levels of p24 expression and viral total intracellular RNA was observed in cells that expressed C-TAT. C-TAT exhibited similar binding affinity to tat37 as V38-TAT and M1-TAT but was catalytically inactive due to the presence of the mutations at the catalytic domain (Figure 2, Table 1). These results suggest that the overall observed inhibition with V38-TAT and M1-TAT was primarily due to targeted cleavage by these ribozymes as opposed to the antisense effect of the ribozyme sequences. Moreover, the ribozyme (V38-TAT) that exhibited higher cleavage activities [(k_cat_/K_m_)^s^] appeared to be more effective in cell culture. These results strongly suggest that increasing the catalytic efficiency of RNase P ribozymes may lead to an improved efficacy in inhibiting HIV-1 gene expression and growth in cultured cells.

It has previously been shown that custom-designed EGS molecules could induce human RNase P to cleave HIV RNA sequence in vitro and could inhibit HIV infection in cultured cells [33], [34]. The approach described in the current study, which uses M1GS ribozymes for blocking HIV infection in human cells, is different from the EGS-based strategy. Ribozyme technology represents an attractive approach for gene inactivation since it exhibits most of the properties of conventional antisense targeting methods and in addition, catalytic and irreversible cleavage of the target RNA. Like aptamers, ribozymes are much less likely than RNAi molecules to saturate cellular factors required for their processing, such as Exportin V, Drosha, or Dicer [8], [9], [10], [16]. Indeed, both hammerhead and hairpin ribozymes have been used for inhibition of HIV replication and infection [9], [17], [18], [44]. The properties and activities of RNase P ribozyme, as well as the simple design of the guide sequence, make M1GS an attractive and unique gene-targeting agent that can be generally used for antiviral as well as other *in vivo* applications. To our knowledge, this study represents the first to use RNase P ribozymes for inhibition of HIV gene expression and replication.

*In vitro* selection has been widely used to generate either new nucleic acid-based catalysts or more efficient variants from known ribozyme molecules [45], [46], [47]. For example, this procedure has been extensively used to generate efficient group I intron, hammerhead, and hairpin ribozyme variants [48], [49], [50], [51]. Using a RNase P ribozyme variant selected from a pool of M1 molecules containing randomized sequences [36], we, in this study, provide the direct evidence that an RNase P variant with increased cleavage activity *in vitro* also exhibits improved efficacy in inhibiting HIV-1 gene expression and growth in cultured cells. Thus, our study provides a direction for the engineering of highly active and effective RNase P ribozyme variants by carrying out selection procedures in order to improve the efficacy of the M1GS-based technology. Further characterization of the cleavage reactions of this as well as other RNase P variants both *in vitro* and in cultured cells should provide insights into the mechanism of how an RNase P ribozyme efficiently cleaves an mRNA substrate and develop guidelines for construction of effective gene-targeting ribozymes.

HIV-1, a member of human retrovirus family, can easily introduce mutations in its genome during replication because of the low fidelity of reverse transcriptase for genome replication [1], [2]. Thus, it is possible that HIV escape mutants with mutations at the targeted *tat* sequence can be generated and become resistant to the ribozymes. In order to achieve effective anti-HIV therapy, multiple ribozymes should be designed to target different regions of the HIV genome that are highly conserved and may encode viral proteins that are essential for HIV replication [7], [8]. These studies will further facilitate the development of M1GS ribozymes for treatment of HIV infection as well as for general gene-targeting applications.
